# Reconstruction of iliac crest with rib to prevent donor site complications: A prospective study of 26 cases

**DOI:** 10.4103/0019-5413.33678

**Published:** 2007

**Authors:** BR Dave, HN Modi, A Gupta, A Nanda

**Affiliations:** Spine Hospital, Mithakhali, Ellisbridge, Ahmedabad, India

**Keywords:** Pathology of dorsal spine, reconstruction of donor site with rib, thoracotomy, tricortical bone graft

## Abstract

**Background:**

The tricortical bone graft from the iliac crest are used to reconstruct the post corpectomy spinal defects. The donor iliac area defect is large and may give rise to pain at donor site, instability of pelvis, fracture of ilium, donor site muscle herniation or abdominal content herniation. Rib removed during thoracotomy was used by us to reconstruct the iliac crest defect.

**Materials and Methods:**

Twenty-six patients who underwent thoracotomy for dorsal spine corpectomy or curettage for various spinal pathologies from June 2002 to May 2004 were included in the study. After adequate decompression the spine was reconstructed by tricortical bone graft from iliac crest and reconstruction of the iliac crest was done with the rib removed for exposure during thoracotomy.

**Results:**

The mean follow up was 15 months. All patients had good graft incorporation which was evaluated on the basis of local tenderness and radiographs. One patient had graft displacement.

**Conclusion:**

The reconstruction of iliac crest by rib is a simple and effective procedure to prevent donor site complications.

Tricortical iliac bone is superior to rib as a graft for reconstruction of the anterior column of the spine. In thoracic spine surgery with anterior approach, during corpectomy or curettage of dorsal spine, usually a large gap is created which is filled with the bone grafts taken from the iliac crest for solid fusion. This graft is usually large and creates a considerable defect in the pelvic bone. Complications related to these defects are many like pain at donor site, instability of pelvis,^1^ fracture of ilium,^2^ donor site muscle herniation or abdominal content herniation [Figure 1].^3^ To prevent donor site complication rib was inserted to restore the normal anatomy of the crest.

**Figure 1:**
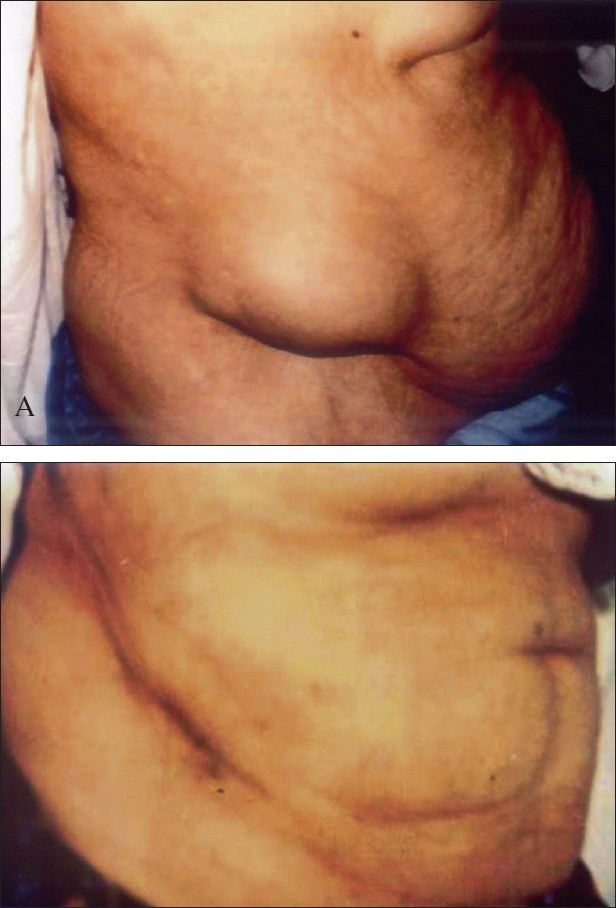
clinical photograph of a patient shows A) incisional hernia from graft donor site. B) after repair of hernia in the same patient

This study was aimed to assess the clinical and radiological results of reconstruction of iliac crest with the help of the excised rib during the anterior approach of thoracic spine.

## MATERIALS AND METHODS

We operated 26 patients from June 2002 to May 2004 with various pathology of thoracic spine. 10 were female and 16 were male. The age ranged from 22 years to 75 years with an average of 38 years. There were 17 patients of tuberculosis of spine, three patients of osteoporotic fracture, two patients of tumor and four patients of traumatic fracture. All these cases were single-level involving the mid to lower dorsal segments (D5 to D12). Since all the cases were single level only one body and two adjacent discs were excised.

**Figure 2:**
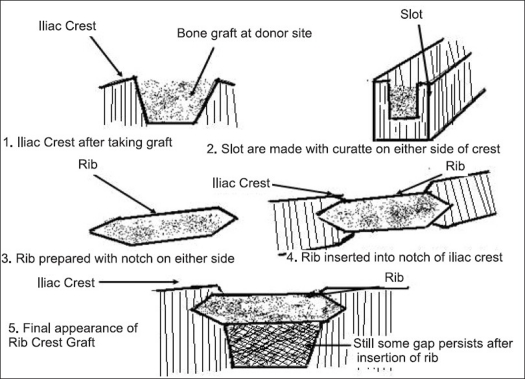
Line diagram shows iliac crest defect after taking out tricortical graft and various stages of iliac crest reconstruction with rib graft

All the patients were operated by transthoracic anterior decompression and reconstruction surgery for dorsal spine pathology. Rib was excised to facilitate better visualization of the affected area and to achieve the proper debridement and decompression. The rib excised was usually one or two levels above the involved segment of the spine in all the cases. The rib to be excised was decided on the basis of per-operative IITV image. The tricortical iliac crest graft of the required size was harvested from the same side of the crest and inserted in the corpectomy site. The length of the tricortical graft usually ranged between 3.5-5 cm. To reconstruct the gap at the iliac crest, the rib which is removed at the time of thoracotomy was used. The rib size used was about 0.5-1 cm more than the length of the tricortical graft. With the small curette slots were made on either end of the donor area. The rib of adequate size was inserted after fashioning it from both ends with convexity outside. The rib graft was beveled from both sides and its length was kept half a centimeter more than the corresponding slot [Figure 2]. It was then hammered in the slot giving it press fit stability without any need of any additional fixation. The muscles erased from the crest were sutured in a single layer over the rib graft.

## RESULTS

**Figure 3:**
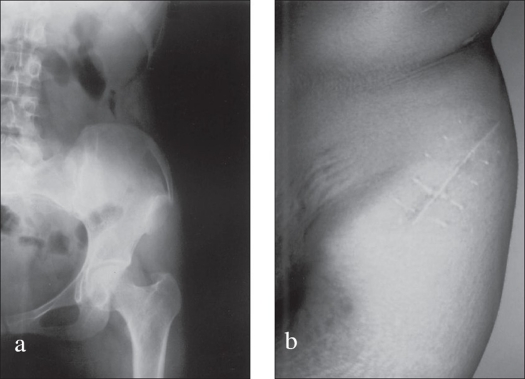
X-ray pelvis a) Showing iliac crest after incorporation of rib graft. clinical photo, b) of the same patient.

Minimum follow-up was three months and maximum of 27 months with mean as 15 months. All the patients were pain-free; the rib graft was well incorporated clinically (no local tenderness) and radiologically. No rib graft complication was recorded. The crest contour was found well maintained [Figure 3].

**Figure 4:**
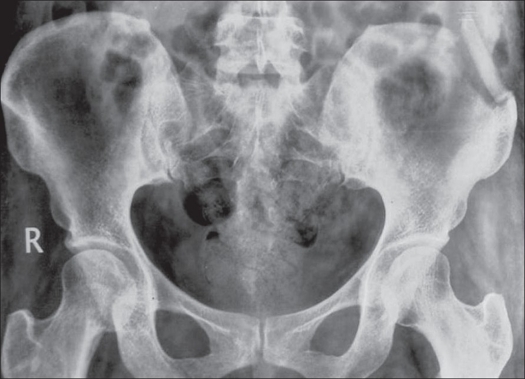
X-ray pelvis showing displaced rib graft

In one patient the rib was displaced from its original position because of partial resorption of the rib which healed eventually [Figure 4]. All other patients were uneventful.

Out of the total 17 cases of Koch's Spine, thirteen of them were paraparetic and four were neurologically normal. There were four cases (all males) of fracture spine all were paraparetic. Three patients (two males, one female) had osteoporotic fractures with no neurological deficit. Two patients (one male, one female) had tumor, out of which the male patient had Plasmacytoma D11 and was paraparetic. The female patient was diagnosed to have a giant cell tumor and was neurologically intact. All these patients improved neurologically and walked after the surgery and had no pain at the graft site.

## DISCUSSION

The vertebral body is exposed by the transthoracic approach in various dorsal spine pathologies. For proper adequate exposure one rib excision may be required to reach the affected vertebra. After corpectomy the anterior fusion is performed by bone graft (iliac or rib), metal cage and bone graft substitutes. Rib harvested is not strong. Bone graft substitutes are expensive and are not without risk of infection. Often the dorsal spine pathology has superadded bacterial infection which makes bone substitute contraindicated. The iliac bone graft provides strength and scaffolding for the bony ingrowth. It leaves a defect in iliac crest. Many studies have shown that the iliac crest graft harvest is not risk-free, with an overall complication rate ranging from 9.4-49%.^4^ These complications were classified to be major and minor.^4^,^5^ The major ones included long-term disabling pain, delayed fracture of ilium,^2^ herniation at harvest site,^3^ deep wound infection etc. Minor complications included superficial infections, seromas and hematomas.

Incisional hernia, although rare, can occur from one month to several years after the original surgery.^3^,^6^ The patients at risk are the elderly, the obese and those with poor musculature. Preventive steps include accurate apposition and suturing of the periosteum and muscular origins as in four-layer hemostatic closure suggested by Banwart *et al.*^4^ Other preventive steps suggested are harvesting of graft only from inner or outer table of crest^3^,^6^ and avoiding massive tricortical grafts.

By bridging the iliac crest defect by rib graft we can create normal-appearing pelvic bone with the piece of rib and without putting the extra incision or exposure. The rib which is grafted into the defect incorporates within three months. Clinically it gives good cosmetic appearance at the reconstruction site and radiologically rib segment shows good integration.

Defino HL and Rodnguez - Fuentes AE^7^ used two pieces of rib; one piece to be put vertical in the defect and over which the other piece to be inserted along the crest. Thus the whole gap of the donor site is filled. Other methods of reconstruction reported in the literature are bioactive ceramic spacer by Manabu *et al.*,^8^ proplast reconstruction by Hochschuler *et al.*^9^ and methylmethacrylate reconstruction by Lubicky *et al*.[10]

In our study, we inserted a single piece of rib in the donor site defect. There was a defect in the iliac wing under the rib graft. However, we did not encounter any complication due to that defect. Partial resorption of rib was found in one patient but it did not affect the clinical or cosmetic result. All the patients walked without any pain and had good graft incorporation.

## CONCLUSION

We recommond use of harvested rib during thoracotomy to reconstruct the iliac crest donor site defect as this procedure is safe, without any complications and no secondary surgery is required.
